# Supplementary material for: Phase separation of TPX2 enhances and spatially coordinates microtubule nucleation
Source: Nat Commun. 2020 Jan 14;11:270. doi: 10.1038/s41467-019-14087-0 (PMC6959270; doi:10.1038/s41467-019-14087-0)
Supplement: Supplementary file 1 — Supplementary Information [file 41467_2019_14087_MOESM1_ESM.pdf]

Supplementary information for

Phase separation of TPX2 enhances and spatially coordinates microtubule nucleation

King et al.

Supplemental Figures

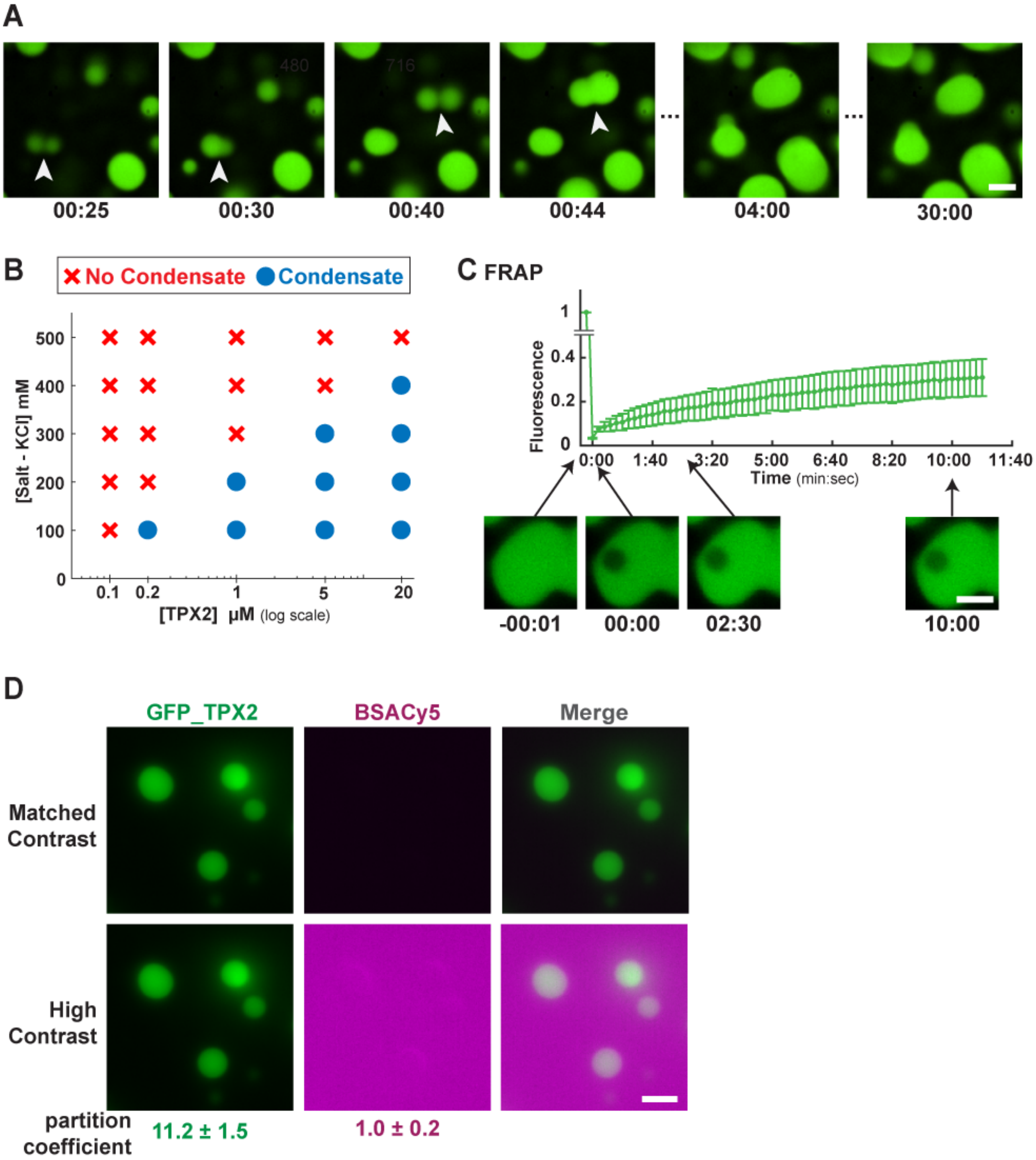

Supplemental Figure 1. TPX2 phase separates into a liquid-like condensate

(A) Confocal microscopy images of GFP-TPX2 condensates falling and fusing in a coverslip-bottomed well. Select frames from time-course shown (Supplemental Movie 1). 00:00 (minutes:seconds) corresponds to when GFP-TPX2 was added to the well. Arrowheads indicate fusion events. GFP-TPX2 at 20µM. Scale bar, 3µm. **(B)** Phase diagram of GFP-TPX2 at indicated salt (mM) and protein (µM) concentrations. Blue circles indicate presence and red crosses indicate absence of condensates**.** **(C)** Fluorescence recovery after photo-bleaching (FRAP) of mCherry-

TPX2 condensates (pseudo-colored green), acquired via confocal microscopy. Mean and SEM of three replicate experiments (error bars) shown. Example images shown immediately before (-00:01) and after photobleaching (00:01). Also shown are two time-points into recovery. Scale bar, 3μm. **(D)** Epifluorescent image of GFP-TPX2 condensates prepared with Cy5-labeled BSA , both at 4 μM. Scale bar, 3μm. In upper panel the contrast is matched to main figure 1C, and enhanced in the lower panel to illustrate the absence of BSA enrichment. Partition coefficient value is the mean with ±1 standard deviation (SD) computed from at least 100 condensates in an experimental set.

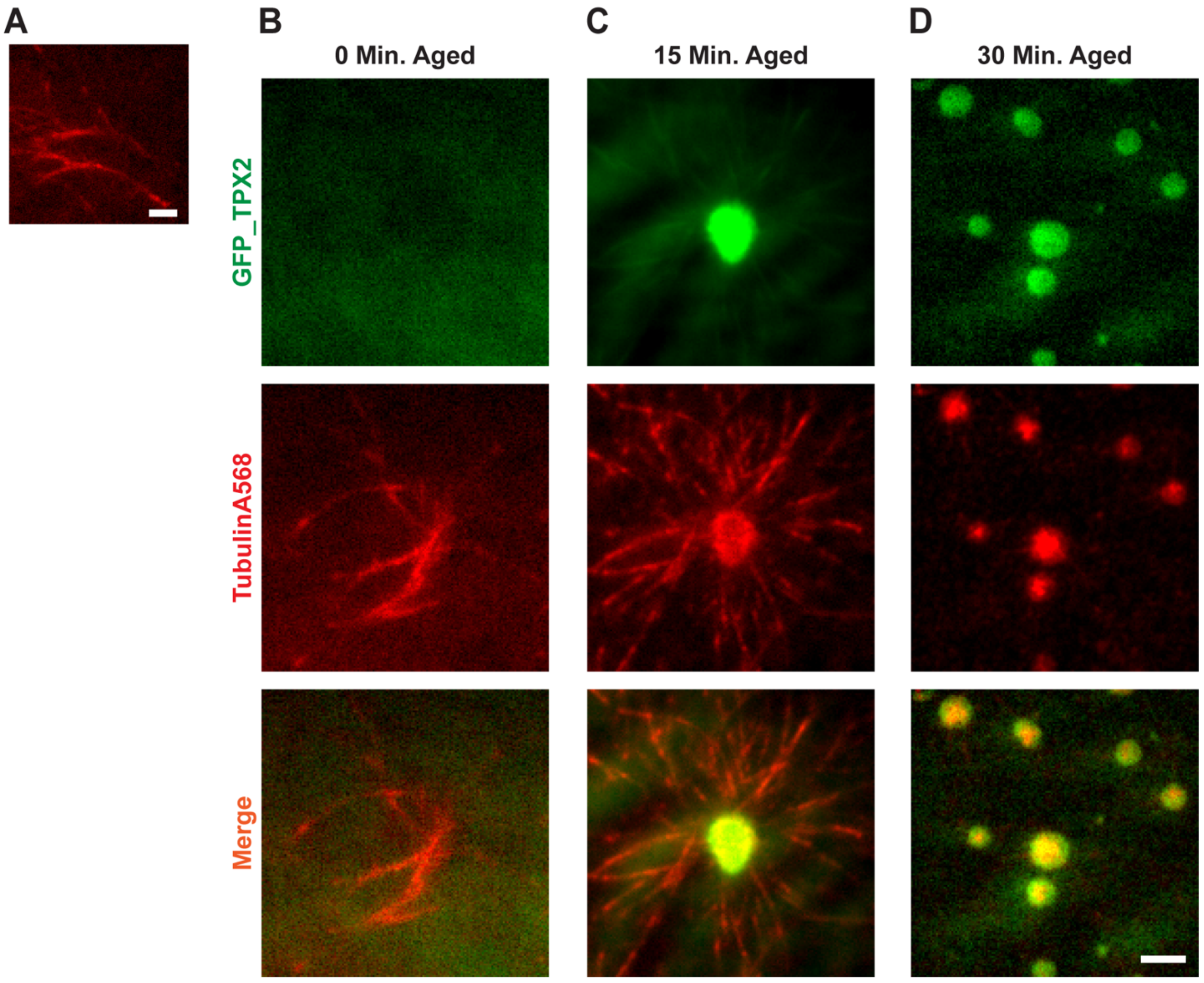

Supplemental Figure 2. TPX2 condensate age affects MT nucleation.

(A) An alternate field from same experiment as Fig. 1G-H and Sup. **(B-D)** Data from Fig. 2B at 20 minutes into the reaction. Scale bar, 3µm. GFP-TPX2 condensates aged for **(B)** 0 minutes **(C)** 15 minutes and **(D)** 30 minutes were overlaid with cytosol containing mono-dispersed Alexa568-labeled tubulin (see schematic in Fig. 1H). Images acquired via oblique TIRF microscopy 20 minutes after sample preparation. GFP-TPX2 and tubulin (Alexa568-labeled) channels and merge shown. TPX2 at 2 µM; scale bar, 3µm.

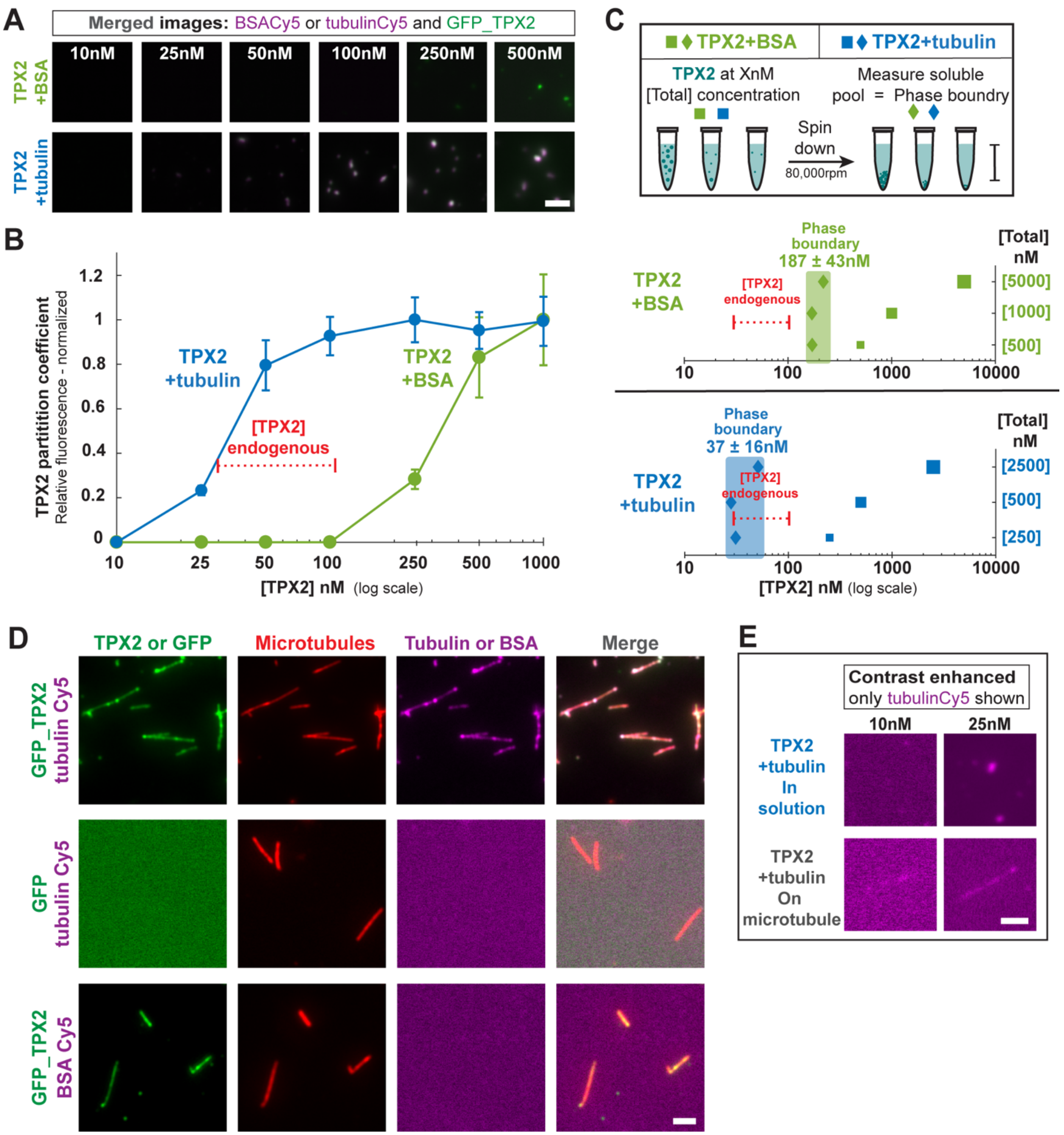

Supplemental Figure 3. The phase boundary of TPX2 is lowered by tubulin, and TPX2-tubulin co-condensates specifically form on microtubules.

(A) Epifluorescent images of TPX2 condensates with BSA and TPX2-tubulin co-condensates *in vitro*. BSA, tubulin, and TPX2 are equimolar at indicated concentrations. Scale bar, 2µm. **(B)** Graph of partition coefficient of GFP-TPX2 normalized to the maximum partition coefficient among the concentrations shown. Mean values shown as circles with ±1 SD shown as error bars for condensates formed in the presence of BSA (green) or tubulin (blue) and plotted as a function

of concentration. At least 100 condensates per concentration were analyzed. Endogenous concentration range of TPX2 (30-100 nM) indicated. **(C)** Schematic of method used to determine soluble concentration of GFP-TPX2 at various total concentrations in the presence of BSA and tubulin. Total concentration (squares) is indicated on the Y-axis and the corresponding soluble pool measurement (diamonds) is indicated on the same plane. Mean soluble pool concentrations of three replicate experiments shown. Phase boundary value is the mean SEM of all soluble pool replicates. **(D)** Oblique TIRF images (larger fields of view than shown in Fig. 2D, F, and I) of pre-formed MTs (stabilized with GMPCPP and labeled with Alexa568) in the presence of GFP-TPX2 and Cy5-labeled tubulin (upper panel), GFP and Cy5-labeled tubulin (middle panel), or GFP-TPX2 and Cy5-lableled BSA (lower panel). Note that tubulin (Cy5-labeled) does not bind to MTs (Alexa568-labeled) unless GFP-TPX2 is present. Contrast is maximized in these images. All proteins at equimolar concentration (100 nM). Scale bar, 3μm. **(E)** Enhanced contrast versions of 10nM and 25nM images shown in Fig. 2F of oblique TIRF images of only Cy5-labeled tubulin condensed with GFP-TPX2. Scale bar, 2μm.

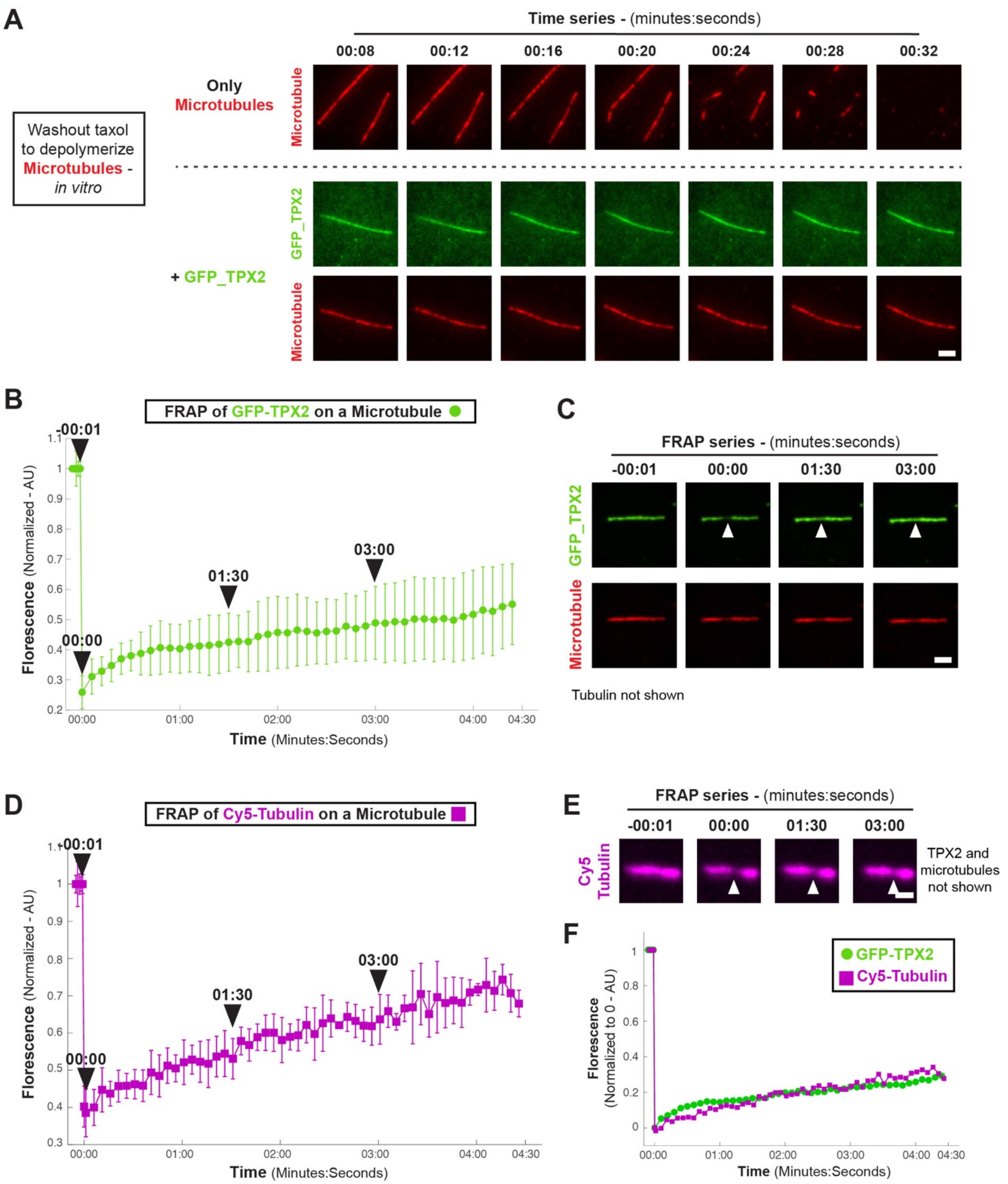

Supplemental Figure 4. TPX2 protects MTs from de-polymerization and TPX2 and tubulin recover from photobleaching on MTs

(A) TIRF images of taxol stabilized Alexa568-labeled MTs (red) after buffer exchange to washout taxol and induce MT de-polymerization. Upper panel is MTs alone and lower panel contains 250nM GFP-TPX2 (green) and Cy5-tubulin (not shown). Time series at intervals of

acquisition (4 seconds) are shown. Scale bar 2μm. **(B)** Graph of Fluorescence Recovery After Photobleaching (FRAP) of GFP-TPX2 on GMP-CPP stabilized Alexa568-labeled MTs. 250nM GFP-TPX2 and Cy5-tubulin (tubulin channel not shown), pre-mixed, were added to a coverslip well containing stabilized MTs (schematized in figure 2H) and reaction was incubated for 5 minutes prior to photobleaching. Recovery curve shows GFP fluorescence in photoactivated area normalized to background accumulation of GFP-TPX2 on nearby MTs. **(C)** Spinning disc confocal images of select time points of GFP-TPX2 (green) and Alexa568-labeled MTs (red) from FRAP experiment (tubulin present but not shown). Scale bar 2μm. **(D)** Graph of FRAP of Cy5-tubulin on GMP-CPP stabilized Alexa568-labeled MTs in presence of GFP-TPX2. Reaction set-up and conditions match panels B and C, however photobleaching laser intensity used was 4X higher and dwell time 10X longer. Recovery curve shows Cy5 fluorescence in photoactivated area normalized to background. **(E)** Spinning disc confocal images of select time points of Cy5-Tubulin (magenta) from FRAP experiment (TPX2 and microtubules present but not shown). Scale bar 1μm. **(F)** Replotted graph of FRAP graphs from panels B and D, GFP-TPX2 and Cy5-tubulin, respectively. Fluorescent values of each FRAP is normalized to 0 to represent relative recovery and allow FRAP profiles to be compared.

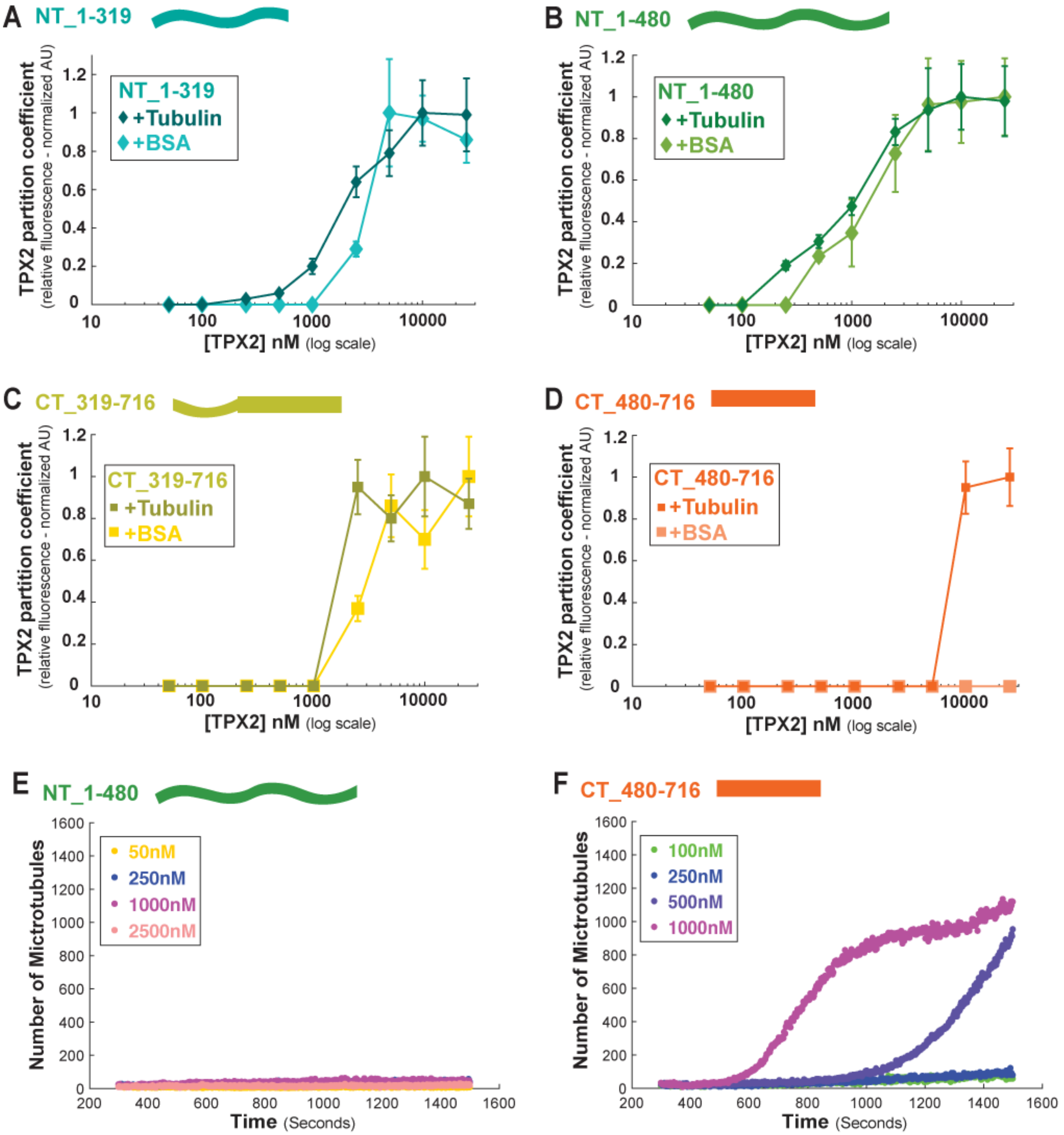

Supplemental Figure 5. Partition coefficients of TPX2 truncation constructs and their MT nucleation ability.
Partition coefficients of GFP_TPX2 in +BSA and +tubulin conditions for the constructs **(A)** NT_1-319, **(B)** NT_1-480, **(C)** CT_319-716, **(D)** CT_480-716. Mean values with ±1 SD as error bars shown. At least 100 condensates per concentration and condition were analyzed. Total number of MTs generated over time for **(E)** NT_1-480 TPX2 and **(F)** CT_480-716 TPX2. Measurements taken at various concentrations of TPX2 (shown in figure).

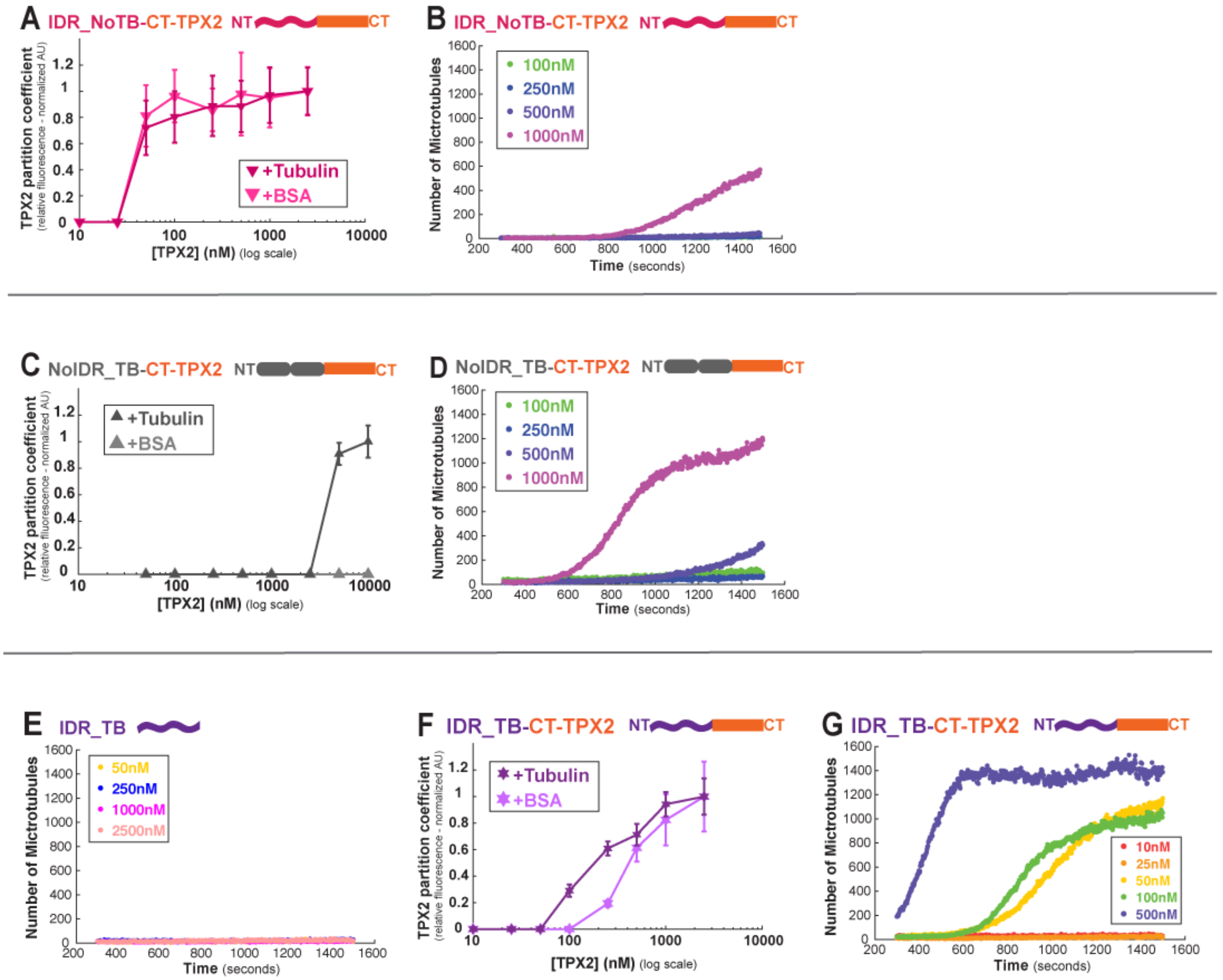

Supplemental Figure 6. Partition coefficients and MT nucleation rate curves of TPX2 chimera constructs

(A) Partition coefficients of GFP_TPX2 in +BSA and +tubulin and **(B)** total number of MTs generated over time for IDR_NoTB-CT-TPX2. **(C)** Partition coefficients of GFP_TPX2 in +BSA and +tubulin and **(D)** total number of MTs generated over time for NoIDR_TB-CT-TPX2. IDR_NoTB-CT-TPX2. **(E)** Partition coefficients of GFP_TPX2 in +BSA and +tubulin and **(F)** total number of MTs generated over time for IDR_TB-CT-TPX2. **(G)** Total number of MTs generated over time for IDR_TB. For partition coefficient graphs mean values with ±1 SD as error bars shown and at least 100 condensates per concentration were analyzed. For both types of graph, measurements were taken at various concentrations of TPX2 and are shown in figure.

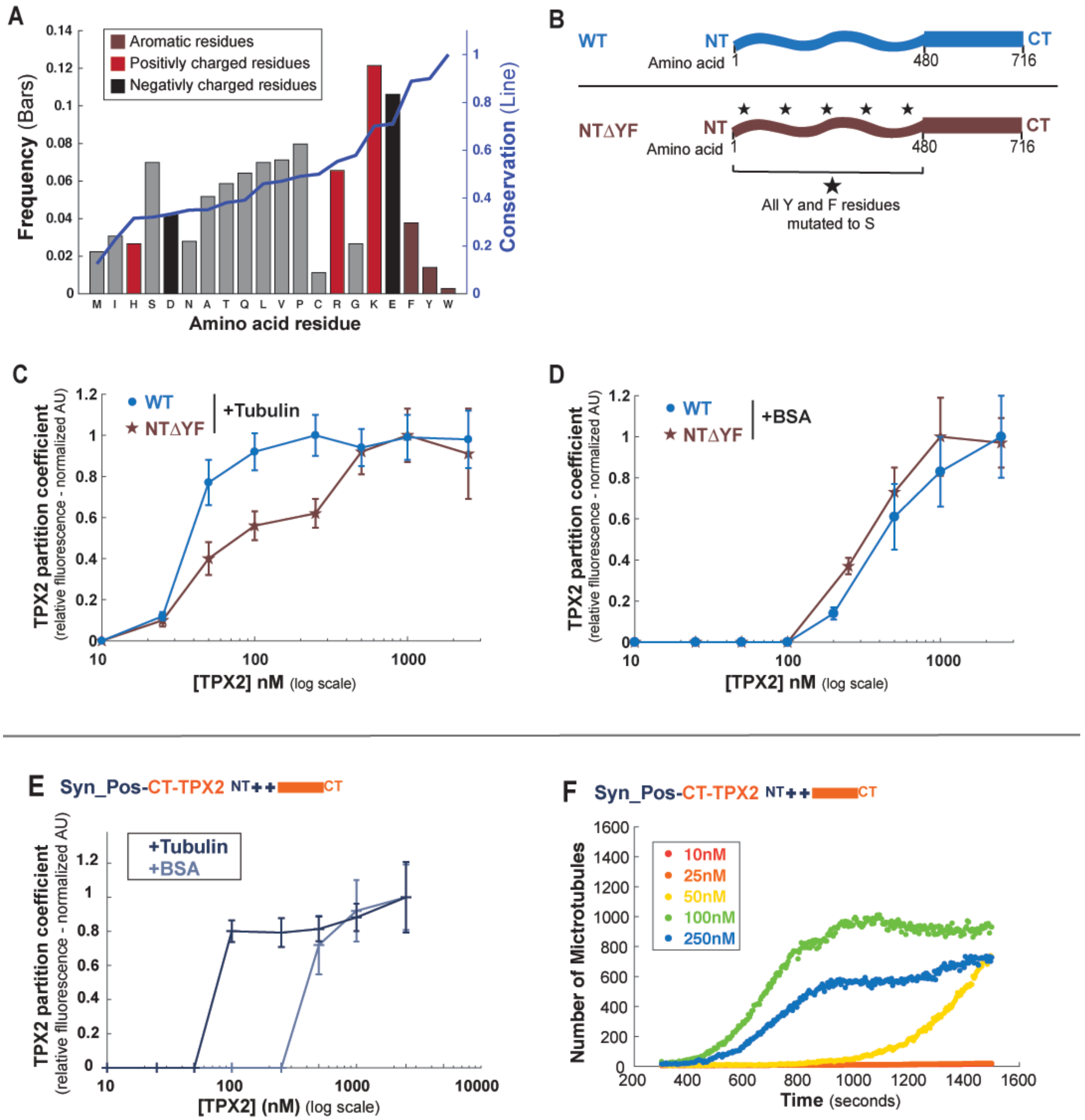

Supplemental Figure 7. Positively charged residues in TPX2 drive co-condensation with tubulin

(A) Graph of amino acids composition of *Xenopus* TPX2. Bars (black borders - left y-axis) displays relative amino acid frequency and line (blue - right y-axis) shows percent conservation of each amino acid type relative to Human TPX2. Amino acids are ordered from least to most conserved and colored (see key). Note that aromatic residues are the most conserved amino acid type, followed by electrostatic residues that are highly conserved and abundant (Lysine –K – being the most abundant). (**B**) Schematic of full length and NTΔYF constructs. Partition coefficients of GFP TPX2 for full length and NTΔYF shown (**C**) +Tubulin and (**D**) +BSA. **(E)** Partition coefficients of GFP_TPX2 in +BSA and +tubulin and **(F)** total number of MTs generated over time for Syn_Pos-CT-TPX2. For partition coefficient graphs mean values with

±1 SD as error bars shown and at least 100 condensates per concentration were analyzed. For both types of graph, measurements were taken at various concentrations of TPX2 and are shown in figure.

Supplementary resources

Name	Primer Sequence
MK37	acctgtacttccagggatccGAAGATACACAGGACACC
MK38	ttagatctggatcttgtacaTCAACACTTAAACCTGTCC
MK39	acctgtacttccagggatccGTGAGCAAGGGCGAGGAG
MK40	ggtggtggcggttctCTTGTACAGCTCGTCCATGC
MK41	gctgtacaagagaaccgccaccaccGAAGATACACAGGACACC
MK42	acctgtacttccagggatccATGGTGAGCAAGGGCGAG
MK43	gtgtatcttcAGAACCGCCACCACCCTT
MK44	tggcggttctGAAGATACACAGGACACC
MK48	gaagatacacaggacaccta
MK49	caaaggagggcacactgttc
MK68	GAATTCCAATTGGAGCTCTCCG
MK71	GGAGGCGGGGGAAGCATG
MK72	gcttcccccgcctccTTTTTCGAACTGCGGGTG
MK73	gccacccgcagttcgaaaaaggaggcgggggaagcGAAGATACACAGGACACC
MK74	gatctggatcttgtacattaTCAACACTTAAACCTGTC
MK75	TAATGTACAAGATCCAGATCTAAGCTTGG
MK76	ACCAGATCCGCTGCTGTG
MK77	atcacagcagcggatctggtGTGAGCAAGGGCGAGGAG
MK78	aggttttcaccaccaccaccACCCTTGTACAGCTCGTC
MK79	GGTGGTGGTGGTGAAAAC
MK80	TCCCTGGAAGTACAGGTTTTC
MK81	gtgaaaacctgtacttccagggaGGTGGTGGCGGTTCTGAAG
MK82	gccgcaatcagactgatggaTCAACACTTAAACCTGTCCGAG
MK83	TCCATCAGTCTGATTGCG
MK84	GTGATGATGATGATGATGG
MK85	agccatcatcatcatcatcacGTGAGCAAGGGCGAGGAG
MK86	gtgtcctgtgtatcttctccctggaagtacaggttttcCTTGTACAGCTCGTCCATGC
MK87	gaaaacctgtacttccagggaGAAGATACACAGGACACC
MK88	agccatcatcatcatcatcacggtggtagcggtagcGTGAGCAAGGGCGAGGAG
MK89	agccatcatcatcatcatcacGTGTCTAAGGGCGAAGAG
MK90	gtgtcctgtgtatcttctccctggaagtacaggttttcATTAAGCTTGTGCCCCAG
MK91	agccatcatcatcatcatcacggtggtagcggtagcGTGTCTAAGGGCGAAGAG
MK92	agccatcatcatcatcatcacGTGAGCAAGGGCGAGGAG
MK93	gtgtcctgtgtatcttctccctggaagtacaggttttcACCCTTGTACAGCTCGTC
MK94	agccatcatcatcatcatcacggtggtagcggtagcGTGAGCAAGGGCGAGGAG

MK95	TTTCTCGAACTGCGGGTG
MK96	cccgcagttcgagaaaCACCATCACCATCACCATG
MK97	gtgtcctgtgtatcttcGGATTGGAAGTACAGGTTTTC
MK98	GAAGATACACAGGACACC
MK99	gacaggtttaagtgttgatgt
MK100	cgagaagttgggggatctggg
MK101	tcaaaggtggaaccagtacag
MK102	attttgctcagtattcacaga
MK103	gctaccgctaccaccATTAAGCTTGTGCCCCAG
MK104	gcacaagcttaatggtggtagcggtagcGACAAAAAGACTATAGTTTGGTTTAGAAGAG
MK105	ccctggaagtacaggttttcgctaccgctaccaccGGTGGCGACCGGTGGATC
MK106	ggtggtagcggtagcGAAAACCTGTACTTCCAGG
MK107	gctaccgctaccaccTCAGGGGGAATATTTTCTG
MK108	aatattccccctgaggtggtagcggtagcGACAAAAAGACTATAGTTTGGTTTAGAAGAG
MK109	tctcagagagccgtctctgggctaccgctaccaccGGTGGCGACCGGTGGATC
MK110	ggtggtagcggtagcCCAGAGACGGCTCTCTGAG
MK111	gctaccgctaccaccACCCTTGTACAGCTCGTC
MK112	gctgtacaagggtggtggtagcggtagcGACAAAAAGACTATAGTTTGGTTTAGAAGAG
MK113	ccctggaagtacaggttttcgctaccgctaccaccGGTGGCGACCGGTGGATC
MK114	ggtggtagcggtagcGAAAACCTGTACTTCCAGG
MK115	GACAAAAAGACTATAGTTTG
MK116	GGTGGCGACCGGTGGATC
MK117	ttgtccatcttcattccctggaagtacaggttttcct
MK118	gatccaccggtcgccaccgaagatacacaggacacctacag
MK119	gatccaccggtcgccaccggaggcggtAGCgaagatacacaggacacctacag
MK120	aggaaaacctgtacttccagggaatgaagatggacaaaaagactatagtttg
MK121	ctgtaggtgtcctgtgtatcttcggtggcgaccggtggatc
MK122	ctgtaggtgtcctgtgtatcttcGCTaccgcctccggtggcgaccggtggatc
MK123	CTTCATACGCGTGGCCGCACACTTAAACCTGTCCGAG
MK124	tctcggacaggtttaagtgtGCGGCCACGCGTATGAAG
MK125	gatctggatcttgtacatcaGGTGGCGACCGGTGGATC
MK126	GATCCACCGGTCGCCACCTGATGTACAAGATCCAGATC
MK127	GTGGCTCCAGCTTGCCATCCGCGCCCGATGGTGGGA
MK128	caccatcgggcgcggATGGCAAGCTGGAGCCAC
MK129	gcttcggaccgggatTCAACACTTAAACCTGTCCGAG
MK130	CTCGGACAGGTTTAAGTGTTGAATCCCGGTCCGAAGCGCG
MK131	GTGGCTCCAGCTTGCCATCCGCGCCCGATGGTGGGA
MK132	caccatcgggcgcggATGGCAAGCTGGAGCCAC
MK133	gcttcggaccgggatTCAGGTGGCGACCGGTGG

MK134	CCACCGGTCGCCACCTGAATCCCGGTCCGAAGCGCG
MK135	tcttgtacatggtggcgaccggtggatccc
MK136	ggtcgccacctgatgtacaagatccagatc
MK137	GCGCTTCATTTCGCAGTCTTTGTCCATgctaccaccGTGATGATGATGATGATGG
MK138	agccatcatcatcatcatcacggtggtagcATGGACAAAGACTGCGAAATGAAGCGC
MK139	tggaagtacaggttttcACCCAGCCCAGGCTTGCC
MK140	GGCAAGCCTGGGCTGGGTGAAAACCTGTACTTCCAGGG
MK141	tccagggagcggccacgcgtatgaag
MK142	tccctggaagtacaggttgtggccgc
MK143	CATCACGGTTCTTCATCTgtgtctaagggcgaa
MK144	AGATGAAGAACCGTGATGgtgatgatgatgatg
MK145	GTTGGGTATAATCGTTTGAGGCCATTCCCTGGAAGTACAGGTTTTC
MK146	aggaaaacctgtacttccagggaATGGCCTCAAACGATTATACCCAAC
MK147	actggggagggtccctccatTCCACGGTCCTGCTGTCC
MK148	GGACAGCAGGACCGTGGAATGGAGGGACCCTCCCCA
MK149	GTATCTTGGGTATCCTCCATTCCCTGGAAGTACAGGTTTTC
MK150	aggaaaacctgtacttccagggaATGGAGGATACCCAAGATAC
MK151	actggggagggtccctccatTTCACGTTGACGGCTACG
MK152	CGTAGCCGTCAACGTGAAATGGAGGGACCCTCCCCA
MK153	atctggatcttgtacatcaaTTCACGTTGACGGCTACG
MK154	CGTAGCCGTCAACGTGAATTGATGTACAAGATCCAGATCTAAGCTTGG
MK155	gaaaacctgtacttccaggg
MK156	tgaatttagggacctcatct
MK157	tgtggcatacgagtggctttAATAACCGGAACCATTTC
MK158	GAAATGGTTCCGGTTATTAAAGCCACTCGTATGCCAC
MK159	gatctggatcttgtacatcaAATAACCGGAACCATTTC
MK160	GAAATGGTTCCGGTTATTTGATGTACAAGATCCAGATCTAAGCTTGG
MK161	tgtggcatacgagtggctttTCCACGGTCCTGCTGTCC
MK162	GGACAGCAGGACCGTGGAAAAGCCACTCGTATGCCAC
MK163	GTCTTTTGTTCAAGTATTCGTCTCCTTCCCTGGAAGTACAGGTTTTC
MK164	aggaaaacctgtacttccagggaAGGAGACGAATACTTGAACAAAAGAC
MK165	tgtggcatacgagtggctttGTAGCGGCCACCTTGAGAC
MK166	GTCTCAAGGTGGCCGCTACAAAGCCACTCGTATGCCAC
MK167	CAGCCTTGCGTTTCTTCGCTCCCTGGAAGTACAGGTTTTC
MK168	aggaaaacctgtacttccagggaGCGAAGAAACGCAAGGCTG
MK169	tgtggcatacgagtggctttCGCTTTGCGTTTTTTCGC
MK170	GCGAAAAAACGCAAAGCGAAAGCCACTCGTATGCCAC
MK171	GTAAACATCTTGGGTGTCCTCTCCCTGGAAGTACAGGTTTTC

MK172	aggaaaacctgtacttccagggaGAGGACACCCAAGATGTTTAC
MK173	tgtggcatacgagtggctttAACCAGGCTCAGCATTTC
MK174	GAAATGCTGAGCCTGGTTAAAGCCACTCGTATGCCAC
MK175	ttgtacatcaAACCAGGCTCAGCATTTC
MK176	gagcctggttTGATGTACAAGATCCAGATCTAAGCTTGGTAC
MK177	ttgtacatcaGTAGCGGCCACCTTGAGAC
MK178	tggccgctacTGATGTACAAGATCCAGATC
MK179	ttgtacatcaTCCACGGTCCTGCTGTCC
MK180	ggaccgtggaTGATGTACAAGATCCAGATCTAAGCTTG
MK181	GTCTTTTGTTCAAGTATTCGTCTCCTCATGCTTCCCCCGCCTCC
MK182	tcggaggcgggggaagcatgAGGAGACGAATACTTGAACAAAAGAC
MK183	agctcctcgcccttgctcacGTAGCGGCCACCTTGAGAC
MK184	GTCTCAAGGTGGCCGCTACGTGAGCAAGGGCGAGGAG
MK185	ttgtacatcaGGGTTTCGGAACAGTGTG
MK186	tccgaaacccTGATGTACAAGATCCAGATCTAAG
MK187	CAAGTATTCGTCTCCTCATGCCTTGAAAGTACAAGTTTTCTC
MK188	gagaaaacttgtactttcaaggcATGAGGAGACGAATACTTG
MK189	gatctggatcttgtacattaTTATTTTTCGAACTGCGG
MK190	CCGCAGTTCGAAAAATAATAATGTACAAGATCCAGATC
MK191	CATCCACTCGCTGTCATCCCCCATTCCCTGGAAGTACAGGTTTTC
MK192	aggaaaacctgtacttccagggaATGGGGGATGACAGCGAGTGGATG
MK193	tgtggcatacgagtggctttAGCTGGAGCCGCCGCAGC
MK194	GCTGCGGCGGCTCCAGCTAAAGCCACTCGTATGCCAC
**Name**	**Notes**
MK37	TPX2_FL
MK38	TPX2_FL
MK39	GFP_TPX2_FL - Gibson - 3 primers
MK40	
MK41	
MK42	mCh_TPX2_FL - Gibson 3 primers
MK43	
MK44	
MK48	TPX2_1bp_F Seq Primer
MK49	TPX2_800bp_F Seq Primer
MK68	Open C-term Rainbow 5'
MK71	Open C-term Rainbow 3'

MK72	Open C-term Rainbow 5' at Str + 3' linker
MK73	TPX2_FL Fwr + 5' Linker
MK74	TPX2_FL Rev
MK75	Open C-term Rainbow 3' at Str
MK76	Open psT50_SHT 5' (US of TEV)
MK77	mCh Fwr
MK78	mCh Rev
MK79	Open psT50_SHT 3' (US of TEV)
MK80	Open psT50_SH_mCh_TEV 5' (DS TEV)
MK81	(link)TPX2 Fwr
MK82	(link)TPX2 Rev
MK83	Open psT50_SH_mCh_TEV 5' (DS TEV)
MK84	iPCR Rev Open psT50 3' to His and 5' to TEV to remove TEV_Fluor
MK85	Fwr Amplify GFP with 5'TEV
MK86	Rev Amplify GFP with 5'TEV
MK87	iPCR Fwr Open psT50 5' of Flu to remove TEV_Fluor
MK88	Fwr Amplify GFP with 5'TEV incoperate linker btw His and GFP
MK89	Fwr Amplify BFP with 5'TEV
MK90	Rev Amplify BFP with 5'TEV
MK91	Fwr Amplify BFP with 5'TEV incoperate linker btw His and BFP
MK92	Fwr Amplify mCh with 5'TEV
MK93	Rev Amplify mCh with 5'TEV
MK94	Rev Amplify mCh with 5'TEV incorperate linker btw His and mCh
MK95	Alt: iPCr RevOpen psT50 3' to Str and 5' to TPX2 to remove HTEV_mCH
MK96	Fwr Amplify His_mCh_TEV from pSP 308
MK97	Rev Amplify His_mCh_TEV from pSP 308
MK98	Alt: iPCr Fwr Open psT50 3' to Str and 5' to TPX2 to remove HTEV_mCH
MK99	Fwr SS Mutagenesis Fix Stopcodon
MK100	Rev SS Mutagenesis Fix Stopcodon
MK101	Fwr SS Mutagenesis Fixmissense at aa50
MK102	Rev SS Mutagenesis Fixmissense at aa50
MK103	Open psT50_SH_BFP_TEV 3' (US TEV)
MK104	Cry2 instert Fwr
MK105	Cry2 instert Rev
MK106	Open psT50_SH_BFP_TEV 5' (DS TEV)
MK107	Open psT50_SH_GFP_TEV 3' (US TEV)
MK108	Cry2 instert Fwr
MK109	Cry2 instert Rev
MK110	Open psT50_SH_GFP_TEV 5' (DS TEV)

MK111	Open psT50_SH_mCh_TEV 3' (US TEV)
MK112	Cry2 instert Fwr
MK113	Cry2 instert Rev
MK114	Open psT50_SH_mCh_TEV 5' (DS TEV)
MK115	Fwr Seq Cry2
MK116	Rev Seq Cry2
MK117	Vec rev Open psT50_SH_GFP_TEV 3' (US TPX2)
MK118	Vec fwr w/o link Open psT50_SH_mCh_TEV 5' (DS TEV)
MK119	Vec fwr w link Open psT50_SH_mCh_TEV 5' (DS TEV)
MK120	Insert Fwr Cry2 WT
MK121	Insert Rev Cry2 WT w/o Linker
MK122	Insert Rev Cry2 WT w/ Linker
MK123	iPCR_rev TPX2_Cry2
MK124	Instert_fwd Cry2
MK125	Instert_rev Cry2
MK126	iPCR_fwd TPX2_Cry2
MK127	iPCR_rev pFastBac Cry2_TPX2
MK128	Instert_fwd SH_GFP_Cry2_T_TPX2
MK129	Instert_rev SH_GFP_Cry2_T_TPX2
MK130	iPCR_fwdpFastBac Cry2_TPX2
MK131	iPCR_rev pFastBac TPX2_Cry2
MK132	Instert_fwd SH_GFP_T_TPX2_Cry2
MK133	Instert_rev SH_GFP_T_TPX2_Cry2
MK134	iPCR_fwdpFastBac TPX2_Cry2
MK135	iPCR_Rev excise TPX2 from GFP_Cry2_TPX2
MK136	iPCR_F excise TPX2 from GFP_Cry2_TPX2
MK137	iPCR_rev SNAP_TPX2_Cry2
MK138	Instert_fwd SNAP
MK139	Instert_rev SNAP
MK140	iPCR_fwd SNAP_TPX2_Cry2
MK141	Fwr Splice BFP or SNAP_T_Cry2
MK142	REv Splice BFP or SNAP_T_Cry2
MK143	Fwr Insert liker H_Link_BFP
MK144	Rev Insert liker H_Link_BFP
MK145	iPCR Rev Open psT50 3' to TEV and 5' to TPX2 aa319 to remove NT319
MK146	Insert_Fwr FUS_IDR
MK147	Insert_Rev FUS_IDR
MK148	iPCR Fwr Open psT50 3' to TEV and 5' to TPX2 aa319 to remove NT319
MK149	iPCR Rev to open psT50_SH_GFP_Tev_TPX2 to remove NT319

MK150	Insert Fwr NT319YFtoS
MK151	Insert Rev NT319YFtoS
MK152	iPCR Fwr to open psT50_SH_GFP_Tev_TPX2 to remove NT319
MK153	Insert Rev NT319YFtoS to replace FL_TPX2 in psT50_SH_GFP_Tev_TPX
MK154	iPCR Fwr to open psT50_SH_GFP_Tev_TPX2 to remove entire TPX2 gene
MK155	Sequencing Primer for Fwr TEV (good fro Fus and NT_319_YFtoS)
MK156	sequecning primer for Rev from TPX2 bp1500
MK157	Insert Rev FL NT480YFtoS Use with MK149 and 150
MK158	Vector Fwr FL NT480YFtoS Use with MK149 and 150
MK159	Insert Rev NT480YFtoS Use with MK149 and 150
MK160	Vector Fwr NT480YFtoS Use with MK149 and 150
MK161	Insert Rev FUS_IDR_a5to7_TPX2 Use with MK147
MK162	Vector Fwr FUS_IDR_a5to7_TPX2 Use with MK146
MK163	iPCR Rev to open psT50_SH_GFP_Tev_TPX2 to remove NT480
MK164	Insert_Fwr BugZ_IDR_ΔN
MK165	Insert_Rev_BugZ_IDR_ΔN
MK166	iPCR Fwr to open psT50_SH_GFP_Tev_TPX2 to remove NT480
MK167	iPCR Rev to open psT50_SH_GFP_Tev_TPX2 to remove NT480
MK168	Insert_Fwr NLS
MK169	Insert_Rev_NLS
MK170	iPCR Fwr to open psT50_SH_GFP_Tev_TPX2 to remove NT480
MK171	iPCR Rev to open psT50_SH_GFP_Tev_TPX2 to remove NT480
MK172	Insert_Fwr NT1to480_Scram
MK173	Insert_Rev_NT1to480_Scram
MK174	iPCR Fwr to open psT50_SH_GFP_Tev_TPX2 to remove NT480
MK175	iPCR Rev to open SH_GFP_Tev_NT1to480_Scram_a5to7TPX2 to remove CTa5to7
MK176	iPCR Fwr to open SH_GFP_Tev_NT1to480_Scram_a5to7TPX2 to remove CTa5to7
MK177	BuGZ_IDR alone Rev
MK178	BuGZ_IDR alone Few
MK179	FUS_IDR alone Rev
MK180	FUS_IDR alone Fwr
MK181	iPCR Rev to open GFP_Tev_HS pSP374
MK182	Fwr Amplify BuGZ_iDR from pSP426
MK183	Rev Amplify BuGZ_iDR from pSP426
MK184	iPCR Fwr to open GFP_Tev_HS pSP374
MK185	ipCR rev to open GFP_TPX2 FL at aa277
MK186	ipCR fwr to open GFP_TPX2 FL at stop

MK187	ipCR rev to open GST Vector
MK188	BuGZ_IDR_fwd (insert)
MK189	BuGZ_IDR_rev (insert)
MK190	ipCR fwr to open GST Vector
MK191	iPCR_Vector_rev AR GFP_TPX2 for TOG12_a57TPX2
MK192	Fwr Amp TOG12_pSP347
MK193	REv Amp TOG12_pSP347
MK194	IPCr_Vector_fwr AR GFP_TPX2 for TOG12_a57TPX2
