# Supplementary material for: Phase separation of TPX2 enhances and spatially coordinates microtubule nucleation
Source: Nat Commun. 2020 Jan 14;11:270. doi: 10.1038/s41467-019-14087-0 (PMC6959270; doi:10.1038/s41467-019-14087-0)
Supplement: Supplementary file 3 — Description of Additional Supplementary Files [file 41467_2019_14087_MOESM3_ESM.pdf]

Description of Additional Supplementary Files

File Name: Supplementary Movie 1
Description: GFP_TPX2 condensates falling and fusing on coverslips. Movie corresponds to Supplemental Figure 1.

File Name: Supplementary Movie 2
Description: Mono-dispersed GFP_TPX2 (green) localizing to emerging MT fan networks that are marked with Cy5-tubulin (red) and the plus-tip tracking protein EB1 (Blue). Reaction in Xenopus egg cytosol treated. Frames were acquired every 10 seconds. Movie corresponds to Fig. 1H.

File Name: Supplementary Movie 3.
Description: GFP_TPX2 and Cy5-tubulin co-condensates in Xenopus egg cytosol treated with nocodazole to prevent microtubule polymerization. Frames were acquired at the fastest possible rate (one frame per 0.1 seconds) but the rapid dynamics of the co-condensates often lead to an offset in the overlap of their signal (merge channel). Movie corresponds to Fig. 1I.

File Name: Supplementary Movie 4
Description: GFP_TPX2 and Cy5-tubulin co-condensates on a stabilized MT in vitro, imaged over time. Microtubule (red) – 1st panel, GFP_TPX2 (green) – 2nd panel, and Cy5-labeled tubulin (magenta) – 3rdpanel channels and merge – last panel. Note that three separate fusion events can be observed. Contrast is optimized. Movie corresponds to Fig. 2H-I.

File Name: Supplementary Movie 5
Description:TPX2-mediated branching MT nucleation in Xenopus meiotic cytosol at indicated concentrations of TPX2. Cy5-labeled tubulin (red) and EB1-mCherry (green) highlight microtubules and growing microtubule plus ends, respectively. Movie corresponds to Fig. 2C-E.

File Name: Supplementary Movie 6
Description:TPX2-mediated branching MT nucleation in Xenopus meiotic cytosol at indicated concentrations of NT_1-480 TPX2 (top row) and CT_480-716 TPX2 (bottom row). Cy5-labeled tubulin (red) and EB1-m Cherry (green) highlight microtubules and growing microtubule plus ends, respectively. Movie corresponds to Fig. 3D and S4E-H.

File Name: Supplementary Movie 7
Description: TPX2-mediated branching MT nucleation in Xenopus meiotic cytosol at indicated concentrations of IDR_NoTB-CT-TPX2 (top row) and NoIDR_TB-CT-TPX2 (bottom row). Cy5-labeled tubulin (red) and EB1-mCherry (green) highlight microtubules and growing microtubule plus ends, respectively. Movie corresponds to Fig. 4C and S5B and D.

File Name: Supplementary Movie 8
Description: TPX2-mediated branching MT nucleation in Xenopus meiotic cytosol at indicated concentrations of IDR_TB-CT-TPX2 (top row) and Syn_Pos-CT-TPX2 (bottom row). Cy5-labeled tubulin (red) and EB1-mCherry (green) highlight microtubules and growing microtubule plus ends, respectively. Movie corresponds to Fig. 4C and S5F and S6F.

File Name: Supplementary Movie 9
Description: TPX2-mediated branching MT nucleation in Xenopus meiotic cytosol at indicated fold excess of importins-α/β. Full-length TPX2 at final concentration of 100nM. Cy5-labeled Tubulin (red) and EB1-m Cherry (green) highlight microtubules and growing microtubule plus ends, respectively. Movie corresponds to Fig. 5B-C and E.
